# Migration and TB disease in a high-resource setting: a descriptive analysis of the RefuScreen-TB cohort

**DOI:** 10.5588/ijtldopen.26.0279

**Published:** 2026-11-13

**Authors:** L. Kübler, L. Olbrich, K. Avsar, M. Steinhauser, U. Behrends, U. von Both, M. Seilmaier, M. Ferko, H. Hoffmann, M. Ahmed, B. Pettinger, K. Held, E. Saathoff, C. Geldmacher, M. Hoelscher, N. Heinrich

**Affiliations:** 1 Institute of Infectious Diseases and Tropical Medicine, LMU University Hospital, LMUMedizin, Ludwig-Maximilians Universität München, Munich, Germany;; 2 Division of InfectiousDiseases and Tropical Medicine, Kepler University Hospital, Linz, Austria;; 3 Division of Infectious Diseases and Tropical Medicine, Kepler University Hospital, Linz, Austria;; 4 Fraunhofer Institute for Translational Medicine and Pharmacology ITMP, Immunology, Infection and Pandemic Research, Munich, Germany;; 5 Asklepios Klinik, Gauting, Germany;; 6 Children’s Hospital, TUM University Hospital, Technical University Munich, Munich, Germany;; 7 Division of Paediatric Infectious Diseases, Hauner Children’s Hospital, University Hospital, LMU Munich, Germany;; 8 Klinikum München Schwabing, Munich, Germany;; 9 SYNLAB Gauting & IML red GmbH, WHO-Supranational Reference Laboratory of Tuberculosis, Munich, Gauting, Germany;; 10 Department of Sociology, LMU Munich, Germany.

**Keywords:** tuberculosis, Germany, migrants, refugees, risk factors, screening

## Abstract

**BACKGROUND:**

Refugees and migrants are disproportionately affected by TB and comprise most diagnoses in low-incidence Western European countries. We describe TB disease characteristics and migration patterns among individuals investigated for TB in Munich, Germany.

**METHODS:**

This retrospective, descriptive analysis used data from RefuScreen-TB, a prospective diagnostic validation study conducted between 2017 and 2019, recruiting children and adults with presumptive TB disease or recent TB contact. Diagnostic workup included microbiological testing and demographic, medical, and travel history. Participants were classified as confirmed, unconfirmed, or no TB.

**RESULTS:**

Of 343 recruited individuals, 284 had a classifiable TB status and were included in the analysis. TB disease was diagnosed in 62.3%, with 79.7% microbiologically confirmed. Participants included German (23.6%), European Union (EU) (12.0%), and non-EU citizens (64.4%), of whom 63.4% were refugees. Most TB disease (68.9%) was detected through passive case finding rather than active case finding. Refugees originated from East/West Africa (41.0%/33.3%) or Asia (25.6%); 61.5% reported stays in informal/formal refugee camps and 30.8% in detention; 72.2% of those detained had TB disease.

**CONCLUSIONS:**

TB screening and care in Germany should account for migration-related exposures and maintain diagnostic vigilance beyond initial reception, particularly among migrants and refugees with prolonged or hazardous migration journeys.

TB remains one of the leading causes of death worldwide, with an estimated one quarter of the world’s population infected with Mycobacterium tuberculosis (MTB).^1,2^ Delayed diagnosis and treatment initiation remain major challenges for TB control and contribute substantially to ongoing transmission.^3^ To improve case detection, the World Health Organization recommends systematic screening of high-risk populations, including refugees and asylum seekers.^4^

In the European Union and the European Economic Area (EU/EEA), TB remains a public health problem despite overall low incidence rates.^5^ Notification rates differ considerably across countries,^6^ with certain populations being especially vulnerable and exposed to structural risk factors.^7^ Refugees and asylum seekers are considered particularly vulnerable due to factors such as origin from high-incidence countries, overcrowded living conditions, limited access to health care, and physical and psychosocial stress during migration.^8^ German law mandates TB screening in recently arrived asylum seekers.^9^ Several studies suggest that a substantial proportion of TB cases are not detected during entry screening but are diagnosed months or years after arrival, frequently through passive case finding following symptom presentation.^10,11^ Despite the importance of migrant populations for TB control in low-incidence countries, data on the demographic, migration-related, and clinical characteristics of individuals undergoing TB evaluation remain limited.^8,12^ A better understanding of these characteristics and associated risk factors may help inform targeted screening strategies, improve case detection, and support the interpretation of diagnostic findings in this population.

This is a retrospective, descriptive analysis of data collected within RefuScreen-TB, a prospective study originally designed to evaluate novel TB diagnostic approaches. As secondary analysis, it was not designed to test a specific a priori hypothesis; our objective was to describe the demographic, migration-related, and clinical features of participants who underwent TB evaluation within this cohort, with particular attention to TB-related risk factors and disease characteristics among persons born outside Germany.

## METHODS

This was a retrospective, descriptive analysis of data collected within RefuScreen-TB, a prospective diagnostic validation study conducted in Munich, Germany, between July 2017 and May 2019, which aimed to evaluate novel TB diagnostic approaches. Eligible participants were children and adults of any age and nationality presenting with presumptive TB disease (clinical symptoms and/or radiological findings suggestive of TB) or recent known contact with a person with TB, who were investigated at one of four participating hospitals in greater Munich: two adult (Klinikum München Schwabing, Munich, Germany; Asklepios Klinik Gauting, Germany) and two paediatric centres (Children’s Hospital, TUM University Hospital; Division of Paediatric Infectious Diseases, Hauner Children’s Hospital, University Hospital, LMU Munich). Participants currently receiving TB treatment, or who have received TB treatment for more than 30 days within the previous 3 months, were excluded.

The diagnostic workup was determined by the treating clinicians, and procedures and results were recorded in study forms. Detailed demographic, medical, and migration-related information was collected using standardised questionnaires (Supplementary Data 1).

### Study procedures

Specimen collection for TB microbiology included respiratory samples and others, depending on the treating physician’s discretion, which were tested using both solid (Löwenstein–Jensen medium; LJ) and liquid culture (fully automated BACTEC Mycobacterial Growth Indicator Tube system; BBL^TM^ MGIT^TM^ 960, Becton Dickinson Microbiology Systems, Sparks, MD, USA; MGIT), followed by speciation testing. Real-time PCRs (RT-PCRs) targeting the MTB complex were conducted, and a subset of samples underwent additional testing with the GeneXpert MTB/RIF Ultra® (Cepheid, Sunnyvale, CA; Ultra). Further investigations included haematology, interferon-gamma release assay/tuberculin skin test (IGRA/TST), and radiology. Ralph score was used to describe the extent of disease in chest X-ray (CXR).^13^

### Study definitions

Participants were classified as refugees if they completed the migration questionnaire and as migrants if they (first generation) or their parents (second generation) were born outside Germany. The latter also included participants with German citizenship. Migration routes were defined based on recent migration and country of origin: i) Asian route, ii) East African route, and iii) West/North African route, with these regions being modified versions of those used by the United Nations.^14^

TB disease was categorised as confirmed TB (PCR and/or culture), unconfirmed TB (clinical diagnosis), or no TB. Diagnostic classification was determined by two independent clinicians as part of an Endpoint Review Committee (see Supplementary Data Figure 1). Participants classified as unclassifiable were excluded from further analyses.

### Data analysis

Data were analysed using Stata version 16.1. Continuous variables are summarised using mean and standard deviation (SD) if normally distributed or median and interquartile range (IQR) if not. Categorical values are presented as counts and percentages. Poisson regression analyses with robust variance estimates, rather than logistic regression, was used to calculate risk ratios (RRs) for associations between clinical or demographic characteristics and diagnostic outcomes (TB disease vs. no TB) in the refugee cohort, because of the high prevalence of the outcome in this cohort, which would otherwise lead logistic regression to overestimate the relative risk.^15^ Variables with *P* < 0.1 in univariable analysis were included in multivariable models. For multivariable regression and comparative analyses, RRs and 95% confidence interval and *P* values were reported. Collinearity among risk factors was assessed using the variance inflation factor.

### Ethical statement

The study was performed in accordance with the study protocol and declaration of Helsinki. The study was approved by the Ethics Committee of the Faculty of Medicine at LMU Munich (20-275-V). Written informed consent was obtained prior to any study procedures; for minors, this was provided by a parent or legal guardian, with additional assent from children ≥7 years. Unaccompanied minor refugees aged ≥14 years could be enrolled if they were interested in participating and signed the assent form; oral consent was obtained from their legal guardian.

## RESULTS

Between July 2017 and May 2019, 343 eligible participants were recruited; 59 (17.2%) could not be assigned a definitive TB status and were excluded, leaving 284 (82.8%) for analysis. Of these, 23.6% were German, 12.0% EU, and 64.4% non-EU citizens (Table 1). Sex and age distribution differed by citizenship group: non-EU and EU citizens were predominantly male (73.7%) and younger (median 27.0 years) than German participants, who were more often female and older (median 51.0 years; Table 1). Of 43 children recruited, 34.9% were under 2 years (Supplementary Data Table 1).

**Table 1.** Demographic characteristics at baseline by citizenship.

		German citizen (n = 67)	EU citizen (n = 34)	Non-EU citizen (n = 183)	All (n = 284)
Sex					
Female	n (%)	35 (52.2)	9 (26.5)	48 (26.2)	92 (32.4)
Male	n (%)	32 (47.8)	25 (73.5)	135 (73.8)	192 (67.6)
Age category					
Age in years	Median (IQR)	51.0 (10.0–69.0)	42.0 (27.0–48.0)	27.0 (21.0–36.0)	30.0 (21.0–46.0)
<18 years	n (%)	18 (26.9)	4 (11.8)	21 (11.5)	43 (15.1)
≥18 years	n (%)	49 (73.1)	30 (88.2)	162 (88.5)	241 (84.9)
Migrant status**^A^**					
Migrant	n (%)	21 (31.3)	34 (100.0)	67 (36.6)	122 (43.0)
Refugee	n (%)	1 (1.5)	0 (0.0)	116 (63.4)	117 (41.2)
Time in Germany (years)	Median (IQR)	19.0 (4.0–60.0)	4.0 (2.0–9.0)	3.0 (2.0–5.0)	3.0 (2.0–13.0)
Reason for testing**^B^**					
Symptoms	n (%)	46 (73.0)	27 (79.4)	118 (65.6)	191 (68.9)
Contact	n (%)	15 (23.8)	4 (11.8)	18 (10.0)	37 (13.4)
Admission examination	n (%)	1 (1.6)	1 (2.9)	30 (16.6)	32 (11.6)
Symptoms and contact	n (%)	1 (1.6)	2 (5.9)	4 (2.2)	7 (2.5)
Symptoms, admission examination	n (%)	0 (0.0)	0 (0.0)	10 (5.6)	10 (3.6)
TB status					
Confirmed TB	n (%)	21 (31.3)	22 (64.7)	98 (53.6)	141 (49.6)
Unconfirmed TB	n (%)	7 (10.4)	6 (17.6)	23 (12.6)	36 (12.7)
No TB	n (%)	39 (58.2)	6 (17.6)	62 (33.9)	107 (37.7)

EU = European Union; IQR = interquartile range.

A Migrant = participants whose birth country was not Germany (first generation) or whose parents were born outside of Germany (second generation); refugee = participants who completed our migration questionnaire.

B Totals might differ due to missing data.

Participants originated from 54 countries, mostly from Germany (23.6%, 67/284), followed by Somalia (9.2%, 26/284), Nigeria (8.8%, 25/284), Romania (8.1%, 23/284), and Eritrea (8.1%, 23/284). Out of all participants, 43.0% (122/284) were migrants; of these, 16.4% (20/122) had German, 27.9% (34/122) EU, and 54.9% (67/122) non-EU citizenship. In total, 41.2% (117/284) were classified as refugees, of whom 99.1% (116/117) were non-EU citizens. Because diagnostic evaluation occurred at the time of presentation or screening, time since arrival in Germany (Table 1) closely approximates the time between arrival and diagnosis for most participants.

Most individuals with TB disease were identified through passive case finding based on symptom presentation (68.9%, 191/277). A smaller number were detected through active case finding (contact tracing or admission examination before entry into mass-accommodations). Among children, TB disease was more frequently identified through contact tracing (Supplementary Data Table 1).

### TB testing and characteristics

A total of 3,169 samples for TB microbiological testing were collected: 79.5% (2,520/3,169) sputum or other bronchial secretion, 11.3% (359/3,169) urine, 3.4% (109/3,169) biopsy, and 5.4% (170/3,169) other sample types. Of all MGIT samples, 53.7% (509/948) tested positive, compared with 41.4% (450/1,086) of LJ samples and 34.5% (286/830) of PCR samples. Based on microbiological and clinical findings, 62.3% (177/284) of participants were diagnosed with TB disease, of whom 79.7% were microbiologically confirmed by PCR and/or culture; disease site and treatment initiation by citizenship are shown in Table 2, with extra-pulmonary TB (EPTB) and multi-organ TB predominantly observed in non-EU citizens. Sites of extra-pulmonary involvement included especially lymph nodes (44.4%; 20/45), bone or spinal (20.0%; 9/45), urogenital (6.7%; 3/45), cerebral (6.7%; 3/45), or pleural (6.7%; 3/45) manifestations. One participant refused treatment.

**Table 2.** TB status: TB findings in participants with TB disease (confirmed or unconfirmed TB) by citizenship.

		German citizen (n = 28)	EU citizen (n = 28)	Non-EU citizen (n = 121)	All (n = 177)
TB confirmation status					
Confirmed TB	n (%)	21 (75.0)	22 (78.6)	98 (81.0)	141 (79.7)
Unconfirmed TB	n (%)	7 (25.0)	6 (21.4)	23 (19.0)	36 (20.3)
TB microbiological findings					
LJ positive	n (%)	17 (60.7)	17 (60.7)	69 (57.0)	103 (58.2)
MGIT positive	n (%)	21 (75.0)	22 (78.6)	97 (80.2)	140 (79.1)
PCR positive	n (%)	15 (53.6)	18 (64.3)	71 (58.7)	104 (58.8)
Ultra positive	n (%)	11 (39.3)	11 (39.3)	37 (30.6)	59 (33.3)
TB immunological testing					
TST positive	n (%)	3 (10.7)	3 (10.7)	1 (0.8)	7 (4.0)
IGRA positive	n (%)	14 (50.0)	21 (75.0)	96 (79.3)	131 (74.0)
TB disease site					
Pulmonary TB	n (%)	24 (85.7)	21 (75.0)	87 (71.9)	132 (74.6)
Extra-pulmonary TB	n (%)	1 (3.6)	1 (3.6)	15 (12.4)	17 (9.6)
Pulmonary and extra-pulmonary TB	n (%)	3 (10.7)	6 (21.4)	19 (15.7)	28 (15.8)
TB treatment initiation					
Drug sensitive TB	n (%)	27 (96.4)	26 (92.9)	115 (95.0)	168 (94.9)
Drug resistant TB	n (%)	0 (0.0)	2 (7.1)	6 (5.0)	8 (4.5)

EU = European Union; IGRA = interferon-gamma release assay; LJ = Löwenstein–Jensen medium; MGIT = Mycobacterial Growth Indicator Tube; PCR = polymerase chain reaction; TST = tuberculin skin test.

In those younger than 18 years (n = 43), 30.2% (13/43) had TB disease, 46.2% (6/13) of whom were confirmed microbiologically. All except for one with multi-organ TB (7.7%, 1/13) had pulmonary TB (PTB) (92.3%, 12/13) (Supplementary Data Tables 1–2). Children with TB disease were markedly older (confirmed TB, n = 6, mean 15 [SD 1.7]; unconfirmed TB, n = 7, mean 8.3 [SD 7.4]) compared to those without TB (n = 30, mean 4.8 [SD 5.0]).

TB disease was diagnosed in 46.5% (40/86) of individuals identified through active case finding (contact tracing and/or admission examination with or without symptoms), of whom 27.9% (24/86) were microbiologically confirmed, compared with 70.2% (134/191) and 59.7% (114/191), respectively, among symptomatic participants (Supplementary Data Table 3). Of 25 children under 5, 84.0% (21/25) were tested for TB due to contact tracing and 4.0% (1/25) during admission examination; only 16.0% (4/25) had TB disease, and none microbiologically confirmed.

### Risk factors of TB

Demographic and clinical characteristics differed considerably between groups (Supplementary Data Tables 4–7). Overall, co-morbidities were common, whereas HIV-co-infection was infrequent (2.1%, 6/284; Table 3). Cough was the most frequently reported symptom at baseline (55.3%; 157/284), with TB disease in 68.8% (108/157) of affected participants. Weight loss was recorded in 32.7% (93/284) of participants, of whom 69.9% (65/93) had TB disease. 23.8% (51/214) of participants were underweight (body mass index [BMI] <18.5); among these, 41.2% (21/51) had TB disease.

**Table 3.** Clinical characteristics and risk factors by TB status in the overall cohort.

		TB disease (n = 177)	No TB (n = 107)	All (n = 284)
Citizenship				
Germans	n (%)	28 (15.8)	39 (36.4)	67 (23.6)
EU-citizen	n (%)	28 (15.8)	6 (5.6)	34 (12.0)
Non-EU-citizen	n (%)	121 (68.4)	62 (57.9)	183 (64.4)
CXR findings				
Ralph score	Median (IQR)	10.0 (5.0–32.5)	5.0 (0.0–15.0)	8.0 (3.0–30.0)
CXR finding abnormal (not specified)	n (%)	147 (83.1)	61 (57.0)	208 (73.2)
Symptomology				
Cough	n (%)	108 (61.0)	49 (45.8)	157 (55.3)
Haemoptysis	n (%)	22 (12.4)	10 (9.3)	32 (11.3)
Fever	n (%)	32 (18.1)	16 (15.0)	48 (16.9)
Loss of weight	n (%)	65 (36.7)	28 (26.2)	93 (32.7)
Other laboratory findings				
CRP (mg/l)	Median (IQR)	15.7 (3.8–45.9)	1.5 (1.0–8.0)	6.9 (1.5–32.4)
CRP ≥ 5mg/l	n (%)	121 (68.8)	29 (29.0)	150 (54.3)
Vitamin D levels in ng/ml	Median (IQR)	9.0 (6.0–17.0)	13.5 (9.0–22.0)	10.0 (6.0–20.0)
Vitamin D deficiency (<10 ng/ml)	n (%)	84 (47.5)	22 (20.6)	106 (37.3)
TB risk factors				
Previous TB	n (%)	11 (6.2)	17 (15.9)	28 (9.9)
TB contact	n (%)	57 (32.2)	36 (33.6)	93 (32.7)
Co-morbidities				
HIV	n (%)	3 (1.7)	3 (2.8)	6 (2.1)
BMI	Mean (SD)	22.1 (4.1)	21.2 (6.0)	21.8 (4.9)
Underweight	n (%)	21 (11.9)	30 (28.0)	51 (23.8)
Chronic respiratory diseases	n (%)	8 (4.5)	15 (14.0)	23 (8.1)
Cardiovascular disease	n (%)	22 (12.4)	10 (9.3)	32 (11.3)
Diabetes	n (%)	8 (4.5)	3 (2.8)	11 (3.9)
Tumour/oncologic disease	n (%)	3 (1.7)	2 (1.9)	5 (1.8)
Hepatitis B	n (%)	4 (2.3)	2 (1.9)	6 (2.1)
Other risk factors				
Mine work	n (%)	2 (1.1)	1 (0.9)	3 (1.1)
Indoor cooking (open fire)	n (%)	19 (10.7)	6 (5.6)	25 (8.8)
Smoking	n (%)	61 (34.5)	35 (32.7)	96 (33.8)
Illegal drugs	n (%)	3 (1.7)	1 (0.9)	4 (1.4)
Alcohol abuse	n (%)	38 (21.5)	22 (20.6)	60 (21.1)

BMI = body mass index; CRP = C-reactive protein; CXR = chest X-ray; EU = European Union; IQR = interquartile range.

Among participants with abnormal CXR findings, 70.7% (147/208) were diagnosed with TB disease. Ralph scores were available for 50.7% (144/284), with a median of 8.0 (IQR 3.0–30.0), being higher in participants with TB disease (median 10.0, IQR 5.0–32.5) compared to those without (median 5.0, IQR 0.0–15.0).

### Refugee cohort

Of the overall cohort, 41.2% (117/284) were refugees, of whom 18.8% (22/117) were female, with a median age of 24.0 years (IQR 20.0–30.0). Most refugees originated from East Africa (41.0%; 48/117), followed by West Africa (33.3%; 39/117), and Asia (25.6%; 30/117). Of all individuals diagnosed with TB disease in this cohort, 46.8% (37/79) were from East Africa, while 26.6% (21/79) each were from West Africa and Asia. Further demographics and information on indicators of integration and vulnerability since arrival in Germany (housing situation, marital status, employment status) are summarised in Table 4.

**Table 4.** Refugee cohort.

		TB disease (n = 79)	No TB (n = 38)	All (n = 117)
Sex				
Female	n (%)	17 (21.5)	5 (13.2)	22 (18.8)
Male	n (%)	62 (78.5)	33 (86.8)	95 (81.2)
Age category				
Age in years	Median (IQR)	23.0 (20.0–28.0)	28.5 (22.0–32.0)	24.0 (20.0–30.0)
<18 years	n (%)	7 (8.9)	3 (7.9)	10 (8.5)
≥18 years	n (%)	72 (91.1)	35 (92.1)	107 (91.5)
Flight routes				
Asian route	n (%)	21 (26.6)	9 (23.7)	30 (25.6)
Asian route, duration (weeks)	Mean (SD)	14.7 (19.3)	40.0 (60.1)	21.9 (36.1)
East African route	n (%)	37 (46.8)	11 (28.9)	48 (41.0)
East African route, duration (weeks)	Mean (SD)	45.8 (56.2)	76.3 (85.7)	54.3 (65.9)
West African route	n (%)	21 (26.6)	18 (47.4)	39 (33.3)
West African route, duration (weeks)	Mean (SD)	58.7 (74.3)	66.3 (81.4)	62.7 (76.0)
Shelter types during migration**^A^**				
Detention centre/prison	n (%)	26 (32.9)	10 (26.3)	36 (30.8)
Hospital/clinic	n (%)	0 (0.0)	2 (5.3)	2 (1.7)
Formal refugee camp	n (%)	37 (46.8)	21 (55.3)	58 (49.6)
Informal refugee camp	n (%)	12 (15.2)	2 (5.3)	14 (12.0)
Homeless shelter	n (%)	13 (16.5)	3 (7.9)	16 (13.7)
Homeless	n (%)	13 (16.5)	5 (13.2)	18 (15.4)
Other type of mass accommodation	n (%)	5 (6.3)	3 (7.9)	8 (6.8)
Mode of travel during migration**^A^**				
Bus/car/truck	n (%)	49 (62.0)	21 (55.3)	70 (59.8)
Boat	n (%)	46 (58.2)	17 (44.7)	63 (53.8)
Train	n (%)	25 (31.6)	7 (18.4)	32 (27.4)
Vulnerability and integration indicators				
Current living conditions				
Own apartment	n (%)	16 (20.3)	4 (10.5)	20 (17.1)
Living with extended family	n (%)	2 (2.5)	1 (2.6)	3 (2.6)
Shared apartment	n (%)	21 (26.6)	4 (10.5)	25 (21.4)
Refugee camp	n (%)	37 (46.8)	28 (73.7)	65 (55.6)
Homeless	n (%)	0 (0.0)	0 (0.0)	0 (0.0)
Other	n (%)	3 (3.8)	1 (2.6)	4 (3.4)
Marital status				
Married	n (%)	19 (24.1)	13 (34.2)	32 (27.4)
Living with partner	n (%)	1 (1.3)	0 (0.0)	1 (0.9)
Single	n (%)	57 (72.2)	20 (52.6)	77 (65.8)
Divorced/separated	n (%)	1 (1.3)	2 (5.3)	3 (2.6)
Widowed	n (%)	0 (0.0)	1 (2.6)	1 (0.9)
NA/unknown	n (%)	1 (1.3)	2 (5.3)	3 (2.6)
Previous employment status				
Student	n (%)	27 (34.2)	5 (13.2)	32 (27.4)
Formally employed	n (%)	13 (16.5)	9 (23.7)	22 (18.8)
Self employed	n (%)	6 (7.6)	1 (2.6)	7 (6.0)
Informally employed	n (%)	11 (13.9)	9 (23.7)	20 (17.1)
Unemployed	n (%)	17 (21.5)	11 (28.9)	28 (23.9)
Retired	n (%)	0 (0.0)	0 (0.0)	0 (0.0)
Taking care of household	n (%)	0 (0.0)	0 (0.0)	0 (0.0)
Other	n (%)	2 (2.5)	0 (0.0)	2 (1.7)
NA/unknown	n (%)	2 (2.5)	2 (5.3)	4 (3.4)
Current employment status				
Student	n (%)	25 (31.6)	7 (18.4)	32 (27.4)
Formally employed	n (%)	12 (15.2)	4 (10.5)	16 (13.7)
Self employed	n (%)	1 (1.3)	0 (0.0)	1 (0.9)
Informally employed	n (%)	4 (5.1)	2 (5.3)	6 (5.1)
Unemployed	n (%)	34 (43.0)	22 (57.9)	56 (47.9)
Retired	n (%)	0 (0.0)	0 (0.0)	0 (0.0)
Taking care of household	n (%)	0 (0.0)	0 (0.0)	0 (0.0)
Other	n (%)	2 (2.5)	1 (2.6)	3 (2.6)
NA/unknown	n (%)	1 (1.3)	1 (2.6)	2 (1.7)

Totals might differ because of missing data.

IQR = interquartile range; SD = standard deviation.

A Multiple responses were possible.

Travel duration differed between routes, with the longest duration observed in the West African route (mean 62.7 weeks; SD 76.0), followed by the East African (mean 54.3 weeks; SD 65.9), and lastly the Asian route (mean 21.9 weeks; SD 36.1). During their travels, most participants reported staying in formal refugee camps (49.6%; 58/117) or detention centres (30.8%; 36/117), or being homeless (15.4%; 18/117). For all groups, TB disease was highly prevalent (informal refugee camps 85.7%, 12/14; formal refugee camps 63.8%, 37/58; detention centres 72.2%, 26/36; homeless shelters 81.3%, 13/16; homeless 72.2%, 13/18). The vast majority of participants incarcerated in detention centres travelled one of the African routes via Libya (97.2%; 35/36).

To explore predictors of TB disease in this cohort, we used univariable and multivariable Poisson regression with robust variance estimates (Supplementary Data Table 8). None of the examined factors (sex, age, flight route, flight duration) were significantly associated with TB disease. However, non-significant trends towards higher risk were observed for certain flight routes.

## DISCUSSION

In this retrospective, descriptive analysis, we characterise the demographic and clinical features of individuals undergoing TB evaluation in a low-incidence, high-resource German setting, focusing on refugees and migrants. TB disease occurred predominantly among those from high-incidence countries and was frequently associated with migration-related vulnerabilities, including prolonged migration, detention, and limited health care access during the journey, providing insight that may inform future TB screening and case detection approaches in migrant populations.

Participants originated from a wide range of countries, with German, Somalian, Romanian, and Eritrean citizens forming the largest groups, in agreement with previous reports from Germany.^16–18^ Refugees and migrants from high-burden countries were disproportionately affected by TB disease, consistent with studies from low-incidence settings.^8,16,19,20^ This likely reflects both the higher TB prevalence in countries of origin and migration-related exposures occurring before, during, and after migration.

Migration-related vulnerabilities, including prolonged travel, overcrowded living conditions, unstable housing, and detention, may increase TB risk.^16,21^ In our cohort, more than two thirds of participants reporting incarceration during migration were diagnosed with TB disease. Most refugees travelling along African routes reported detention in Libya, where poor living conditions and limited health care access have previously been described.^22^ We did not collect detailed data on housing, employment, or health care access after arrival in Germany, so our findings reflect exposures accumulated during migration rather than current conditions in Germany. Although based on small numbers, these findings suggest that detention or similarly hazardous migration journeys may help identify elevated TB risk among individuals presenting for TB evaluation in low-incidence settings, and that pre-arrival exposures, not only post-arrival circumstances, warrant attention in this population.

The proportion of migrant and refugee children in our cohort exceeded that in other European paediatric TB studies.^23–25^ Half of the children were investigated for known TB contact, particularly those under 5 years. Children with TB disease were markedly older than those without, and none of the four young children with TB were microbiologically confirmed. These findings highlight the challenges of paediatric TB diagnosis and the importance of contact tracing and clinical assessment in migrant populations.

Most adults with TB in this cohort were identified following symptom presentation rather than active entry screening, consistent with previous cohort studies and systematic reviews.^26,27^ This contrasts with reports that TB among refugees is frequently identified through active case finding and routine entry screening,^28^ and may reflect both declining refugee arrivals and TB notification rates in Germany during 2017–2019 compared with 2015,^29–31^ and ongoing reactivation risk among those already resident, though hospital-based recruitment may also have contributed. Nevertheless, the finding suggests that a substantial proportion of TB cases continue to be identified outside formal entry screening programmes and highlights the importance of maintaining awareness of TB beyond the initial reception period.

EPTB occurred predominantly among migrants and non-EU citizens, consistent with previous European surveillance data.^32^ Current European screening programmes focus mainly on PTB and latent TB infection, potentially under-addressing extra-pulmonary manifestations.^33^

Our findings should also be considered in the broader context of migration and TB control in Europe. The considerably lower TB yield in the UK’s population-based pre-entry screening cohort (147 per 100,000 person-years after migration) than in our cohort (62.3%) likely reflects recruitment strategy: that cohort was screened systematically before entry, whereas our participants were referred because of clinical suspicion or known TB contact.^16^ Although a substantial proportion of our cohort originated from African and other high-burden countries, migration patterns in Europe continue to evolve. Recent large-scale displacement from Ukraine has renewed attention to TB prevention and care among European refugee populations; a 2024 multi-country survey reported comparatively low TB notification rates among people born in Ukraine (12.0 per 100,000 in 2021; 12.8 in 2022), although over a quarter of notified cases were multidrug- or rifampicin-resistant, underlining the continued need for adapted screening and treatment pathways.^34^ Together with a recent systematic review showing how sensitive the cost-utility of preventive treatment for migrants is to assumptions about TB risk,^35^ our findings underscore the value of cohort-level data for informing population-specific screening and prevention strategies.

This is one of the largest cohorts of individuals investigated for TB in Germany in recent decades, but it has limitations. Diagnostic workup followed local clinical practice rather than a standardised protocol, which may have introduced variability in sampling and workup. Hospital-based recruitment may have biased the sample towards more advanced disease, and small subgroup sizes may limit generalisability and the assessment of risk-factor associations. Disease severity beyond radiological and microbiological findings, treatment outcomes, and post-arrival living conditions were not collected in detail, as these fell outside the scope of the original diagnostic study and represent important areas for future, purpose-designed research.

## CONCLUSION

Data on TB among refugees and migrants in Western Europe remain limited. This study described the demographic, migration-related, and clinical characteristics of individuals undergoing TB evaluation in a low-incidence, high-resource German setting. TB disease was more common among refugees from high-TB-burden countries, particularly those exposed to prolonged migration, detention, or limited health care access during migration, and most cases were identified through passive rather than active case finding. EPTB was also more common among refugees and migrants than among European citizens. Together, these findings suggest that TB screening and care in Germany should account for migration-related exposures and maintain diagnostic vigilance beyond the initial reception period, particularly for migrants and refugees with prolonged or hazardous migration histories.

## Supplementary Material

## References

[1] World Health Organization. Global tuberculosis report. Geneva: WHO, 2021.

[2] Houben RMGJ, Dodd PJ. The global burden of latent tuberculosis infection: a Re-estimation using mathematical modelling. PLOS Med. 2016;13(10):e1002152. 10.1371/journal.pmed.1002152 PMC5079585 27780211

[3] Storla DG, Yimer S, Bjune GA. A systematic review of delay in the diagnosis and treatment of tuberculosis. BMC Public Health. 2008;8:15. 10.1186/1471-2458-8-15 PMC2265684 18194573

[4] World Health Organization. WHO consolidated guidelines on tuberculosis. In: Module 2: screening–systematic screening for tuberculosis disease. Geneva: WHO, 2021. 33822560

[5] European Centre for Disease Prevention and Control and the World Health Organization Regional Office for Europe. Tuberculosis surveillance and monitoring in Europe. Geneva: WHO, 2021.

[6] The World Bank Data. Incidence of tuberculosis (per 100,000 people). Washington, DC, USA: The World Bank, 2022. https://data.worldbank.org/indicator/SH.TBS.INCD (Accessed September 2022).

[7] Hargreaves JR, The social determinants of tuberculosis: from evidence to action. Am J Public Health. 2011;101(4):654-662. 10.2105/AJPH.2010.199505 PMC3052350 21330583

[8] Lonnroth K, Tuberculosis in migrants in low-incidence countries: epidemiology and intervention entry points. Int J Tuberc Lung Dis. 2017;21(6):624-637. 10.5588/ijtld.16.0845 28482956

[9] Bundesamt für Justiz. *Infektionsschutzgesetz - IfSG § 36*. Berlin, Germany: Bundesamt für Justiz.

[10] Weigl JAI. Open flanks in the management of tuberculosis in Germany under the pressure of migration. Eur J Clin Microbiol Infect Dis. 2020;39(4):741-747. 10.1007/s10096-019-03791-w 31848791

[11] Weinrich JM, Yield of chest X-ray tuberculosis screening of immigrants during the European refugee crisis of 2015: a single-centre experience. Eur Radiol. 2017;27(8):3244-3248. 10.1007/s00330-016-4684-9 28050691

[12] Kunst H, Tuberculosis in adult migrants in Europe: a TBnet consensus statement. Eur Respir J. 2025;65(3):2401612. 10.1183/13993003.01612-2024 PMC11883149 39672603

[13] Ralph AP, A simple, valid, numerical score for grading chest x-ray severity in adult smear-positive pulmonary tuberculosis. Thorax. 2010;65(10):863-869. 10.1136/thx.2010.136242 20861290

[14] United Nations Statistics Division. Standard country or area codes for statistical use (M49). New York, NY: United Nations, 2021. https://unstats.un.org/unsd/methodology/m49/ (Accessed 9 September 2021).

[15] Barros AJ, Hirakata VN. Alternatives for logistic regression in cross-sectional studies: an empirical comparison of models that directly estimate the prevalence ratio. BMC Med Res Methodol. 2003;3:21. 10.1186/1471-2288-3-21 PMC521200 14567763

[16] Aldridge RW, Tuberculosis in migrants moving from high-incidence to low-incidence countries: a population-based cohort study of 519 955 migrants screened before entry to England, Wales, and Northern Ireland. Lancet. 2016;388(10059):2510-2518. 10.1016/S0140-6736(16)31008-X PMC5121129 27742165

[17] Robert Koch-Institut. Bericht zur Epidemiologie der Tuberkulose in Deutschland für 2020. Berlin, Germany: Robert Koch-Institute, 2021.

[18] Ronald LA, Demographic predictors of active tuberculosis in people migrating to British Columbia, Canada: a retrospective cohort study. Cmaj. 2018;190(8):E209-e216. 10.1503/cmaj.170817 PMC5826706 29483329

[19] Pareek M, The impact of migration on tuberculosis epidemiology and control in high-income countries: a review. BMC Med. 2016;14:48. 10.1186/s12916-016-0595-5 PMC4804514 27004556

[20] Kaushik N, Post-migration follow-up programme for migrants at increased risk of developing tuberculosis: a cohort study. ERJ Open Res. 2018;4(3):00008-2018. 10.1183/23120541.00008-2018 PMC6043723 30018973

[21] Zenner D, Drivers determining tuberculosis disease screening yield in four European screening programmes: a comparative analysis. Eur Respir J. 2023;62(4):2202396. 10.1183/13993003.02396-2022 PMC10568038 37230498

[22] Kuehne A, Health of migrants, refugees and asylum seekers in detention in Tripoli, Libya, 2018-2019: retrospective analysis of routine medical programme data. PLoS One. 2021;16(6):e0252460. 10.1371/journal.pone.0252460 PMC8177456 34086778

[23] Nordholm AC, The epidemiology and clinical characteristics of pediatric tuberculosis in Denmark 2009-2014 highlights targets for prevention. Pediatr Infect Dis J. 2020;39(11):1007-1011. 10.1097/INF.0000000000002787 32496410

[24] Bennet R, Eriksson M. Paediatric tuberculosis cases increased in Stockholm from 1971 to 2015 following the rising number of children with immigrant backgrounds. Acta Paediatr. 2016;105(12):1480-1486. 10.1111/apa.13629 27740736

[25] Fritschi N, Pediatric tuberculosis disease during years of high refugee arrivals: a 6-year national prospective surveillance study. Respiration. 2021;100(11):1050-1059. 10.1159/000517029 PMC8686709 34325426

[26] Saunders MJ, Active and passive case-finding in tuberculosis-affected households in Peru: a 10-year prospective cohort study. Lancet Infect Dis. 2019;19(5):519-528. 10.1016/S1473-3099(18)30753-9 PMC6483977 30910427

[27] Mhimbira FA, Interventions to increase tuberculosis case detection at primary healthcare or community-level services. Cochrane Database Syst Rev. 2017;11(11):Cd011432. 10.1002/14651858.CD011432.pub2 PMC5721626 29182800

[28] Proença R, Active and latent tuberculosis in refugees and asylum seekers: a systematic review and meta-analysis. BMC Public Health. 2020;20(1):838. 10.1186/s12889-020-08907-y PMC7268459 32493327

[29] Hinz T, Media coverage, fake news, and the diffusion of xenophobic violence: a fine-grained county-level analysis of the geographic and temporal patterns of arson attacks during the German refugee crisis 2015-2017. PLoS One. 2023;18(7):e0288645. 10.1371/journal.pone.0288645 PMC10358929 37471431

[30] Bundesamt für Migration und Flüchtlinge. Migrationsbericht 2019. Nürnberg, Germany: Bundesamt für Migration und Flüchtlinge, 2020. https://www.bamf.de/SharedDocs/Anlagen/DE/Forschung/Migrationsberichte/migrationsbericht-2019.html.

[31] Brodhun B, Bericht zur Epidemiologie der Tuberkulose in Deutschland für 2019. Berlin, Germany: Robert Koch-Institut, 2020, pp. 1-102.

[32] Hayward SE, Extrapulmonary tuberculosis among migrants in Europe, 1995 to 2017. Clin Microbiol Infect. 2021;27(9):1347.e1-1347.e7. 10.1016/j.cmi.2020.12.006 PMC8437049 33352301

[33] Seedat F, How effective are approaches to migrant screening for infectious diseases in Europe? A systematic review. Lancet Infect Dis. 2018;18(9):e259-e271. 10.1016/S1473-3099(18)30117-8 29778396

[34] de Vries G, TB among refugees from Ukraine in European countries. IJTLD Open. 2024;1(4):166-173. 10.5588/ijtldopen.24.0062 PMC11231824 38988409

[35] Robayo SD, Risks and consequences of TB and its prevention in cost-utility analyses among immigrants: a systematic review. IJTLD Open. 2025;2(10):555-562. 10.5588/ijtldopen.25.0355 PMC12517258 41089227

