# Supplementary material for: Migration and TB disease in a high-resource setting: a descriptive analysis of the RefuScreen-TB cohort
Source: IJTLD Open. 2026 Nov 13;3(11):722–9. doi: 10.5588/ijtldopen.26.0279 (PMC13764008; doi:10.5588/ijtldopen.26.0279)
Supplement: Supplementary file 1 [file ijtldopen26-0279_supplementarydata1.pdf]

Supplemental material:

Migration and TB disease in a high-resource setting: a descriptive analysis of the RefuScreen-TB cohort

Supplement 1 (Questionnaire):

<table>
<tr><td>aida_TB</td><td>LMU-IMPH- AIDA-TB</td><td>Questionnaire</td><td>QUES</td><td>LMU KLINIKUM</td></tr>
<tr><td></td><td>Version 3.8 (05-Jul-2017)</td><td>Participant-ID: |__|__| - |__|__|__|__| - |__|</td><td>Page 1 of 9</td><td></td></tr>
</table>

<table>
<tr><td>1.</td><td colspan="3">Sociodemographics</td></tr>
<tr><td colspan="4">General Information – The following questions should be filled in by the interviewer</td></tr>
<tr><td>1.1</td><td>Interviewer's name</td><td></td><td>______________________________</td></tr>
<tr><td>1.2</td><td>Date of Interview</td><td></td><td>|__|__| . |__|__|__| . |__|__|__|__|
Day Month Year</td></tr>
<tr><td>1.3</td><td>Participant's ID</td><td></td><td>|__|__| - |__|__|__|__| - |__|</td></tr>
<tr><td>1.4</td><td>Name of the Study Site:</td><td>☐
☐
☐
☐
☐
☐</td><td>01 – Abt. für Infektions- und Tropenmedizin (TR)
02 - Asklepios Fachkliniken München-Gauting (AG)
03 - Haunersches Kinderspital (HK)
04 - Kinderklinik München Schwabing (TU)
05 - Städtisches Klinikum München Schwabing (KS)
Other, specify:______________________</td></tr>
<tr><td>1.5</td><td>Is the participant an adult, minor or child?</td><td>☐
☐
☐</td><td>Adult (≥18 yrs)
Minor (14 - <18 yrs)
Child (< 14 yrs)</td></tr>
<tr><td>1.6</td><td>Gender of participant</td><td>☐
☐</td><td>Male
Female</td></tr>
<tr><td colspan="4">Start of Interview – The following questions should be asked to the participant.
In case of a child participant, the question needs to be answered with information on the child, not on the caretaker.</td></tr>
<tr><td>1.7</td><td>When was the participant born
If even year of birth if unknown, estimate</td><td></td><td>|__|__| . |__|__|__| . |__|__|__|__| (1)
Day Month Year</td></tr>
<tr><td>1.8</td><td>Residential status in Germany</td><td>☐
☐
☐
☐
☐
☐
☐
☐</td><td>Asylum seeker (without registration)
Asylum seeker (with registration)
Undocumented migrant (illegal)
Residency permit (temporary or permanent)
Immigration as dependent family ("Familiennachzug")
German/EU citizen
Other, specify ________________
Unwilling to disclose</td></tr>
<tr><td>1.9</td><td>Nationality
If necessary, state with number according to list; if other than stated on the list insert 999 and specify.
More than one entry allowed in case of multiple nationalities.</td><td>☐
☐
☐
☐
☐
☐
☐
☐
☐
☐</td><td>German
Syrian
Somali
Afghan
Iranian
Eritrean
1. Other, specify country code |__|__|__|
2. Other, specify country code |__|__|__|
3. Other, specify ________________
Unwilling to disclose</td></tr>
</table>

<table>
<tr><td>aida_TB</td><td>LMU-IMPH- AIDA-TB</td><td>Questionnaire</td><td>QUES</td><td>LMU KLINIKUM</td></tr>
<tr><td></td><td>Version 3.8 (05-Jul-2017)</td><td>Participant-ID: |__|__| - |__|__|__|__| - |__|</td><td>Page 2 of 9</td><td></td></tr>
</table>

<table>
<tr><td></td><td>a) Country of birth?
State with number according to list; if other than stated on the list insert 999 and specify.</td><td>☐
☐
☐
☐</td><td>Germany
Other, specify country code |__|__|__|
Other, specify ________________
Unwilling to disclose</td></tr>
<tr><td></td><td>b) For how many years has the participant been living in Germany?</td><td>
☐</td><td>|__|__|__| (years)
Unwilling to disclose</td></tr>
<tr><td></td><td>c) How often does the participant visit his/her country of birth?</td><td>☐
☐
☐
☐
☐</td><td>Never since immigration
< 1x/year
>= 1x/year
Unwilling to disclose
N/A</td></tr>
<tr><td>1.10</td><td>Where do the participant's father/mother originate from?
More than one entry allowed if paternal and maternal origins are different</td><td>☐
☐
☐
☐
☐
☐
☐
☐
☐
☐
☐</td><td>Identical to nationality (as stated in Q 1.9)
German
Syrian
Somali
Afghan
Iranian
Eritrean
1. Other, specify country code |__|__|__|
2. Other, specify country code |__|__|__|
3. Other, specify ________________
Unwilling to disclose</td></tr>
<tr><td>1.11</td><td>Where did the participant live most recently before coming to Germany
Please state number according to list;</td><td>☐
☐
☐
☐
☐
☐</td><td>Identical to nationality (as stated in Q 1.9)
1. Other, specify country code |__|__|__|
2. Other, specify ________________
Don't know
Unwilling to disclose
N/A</td></tr>
<tr><td>1.12</td><td>What is the City/village of the participant's most recent residence?</td><td>
☐
☐
☐</td><td>______________________
Don`t know
Unwilling to disclose
N/A</td></tr>
<tr><td>1.13</td><td>Current living conditions</td><td>☐
☐
☐
☐
☐
☐
☐
☐
☐
☐</td><td>Living in own/rented apartment or house with next of kin ("Kernfamilie") or alone
Living with extended family (including aunts/uncles, parents/ in case of child participant: grandparents)
Refugee camp
Shared flat
Homeless shelter
Homeless/ living on the street
Other, specify ________________
Unwilling to disclose
N/A
Don't know</td></tr>
</table>

<table>
<tr><td>1.14</td><td>What is the participant's current marital status?</td><td>☐
☐
☐
☐
☐
☐
☐</td><td>Married
Living with partner (cohabitation)
Single
Divorced/separated
Widowed
Unwilling to disclose
Not applicable (child)</td><td></td></tr>
<tr><td>1.15</td><td>What was the participant's previous occupational status before leaving home?
More than one entry allowed.</td><td>☐
☐
☐
☐
☐
☐
☐
☐
☐
☐</td><td>Student
Formally employed
Self-employed
Informally employed
Unemployed
Retired
Taking care of household
Other, specify ________________
Unwilling to disclose
Not applicable (child/German citizen)</td><td></td></tr>
<tr><td>1.16</td><td>What is the participant's current occupational status?
More than one entry allowed.</td><td>☐
☐
☐
☐
☐
☐
☐
☐
☐
☐</td><td>Student
Formally employed
Self-employed
Informally employed
Unemployed
Retired
Taking care of household
Other, specify ________________
Unwilling to disclose
Not applicable (child)</td><td></td></tr>
<tr><td colspan="5">2. Route travelled & countries visited</td></tr>
<tr><td colspan="5">If participant is of German origin, and reports no stays abroad >5 months within last 2 years, tick: N/A: ☐ Then go to Q 3.1</td></tr>
<tr><td colspan="5">Please ensure the patient that her/his data are strictly confidential and will not be accessed by the German Immigration Authorities.</td></tr>
<tr><td>2.1</td><td>Date left home country</td><td>
☐
☐</td><td>|__|__| . |__|__|__| . |__|__|__|__| (1)
Day Month Year
Unwilling to disclose
N/A</td><td></td></tr>
<tr><td>2.2</td><td>Date arrival in Germany</td><td>
☐
☐</td><td>|__|__| . |__|__|__| . |__|__|__|__| (1)
Day Month Year
Unwilling to disclose
N/A</td><td></td></tr>
<tr><td>2.3</td><td>Duration of travel</td><td>
☐
☐</td><td>|__|__|__| (weeks)
Unwilling to disclose
N/A</td><td></td></tr>
</table>

<table>
<tr><td rowspan="13">2.4</td><td rowspan="13">Countries passed en route
Please state country number from list in chronological order (most recent last) and duration in weeks
Multiple answers per country allowed.
Transit if <2 weeks, stay if >2 weeks
In case of >10 entries, re-print page.</td><td>☐</td><td colspan="4">Unwilling to disclose</td></tr>
<tr><td></td><td>Country</td><td>City (if applicable)</td><td></td><td>Duration of journey until next stop</td></tr>
<tr><td>0</td><td colspan="2">|__|__|__|
(Home country)</td><td>Departure via* |__|, |__|, |__|, |__|</td><td>|__|__|__| (weeks)</td></tr>
<tr><td>1</td><td>|__|__|__|</td><td>________________</td><td>☐ Transit
☐ Stay → duration |__|__|__| (weeks)
→ accommodation** |__|, |__|, |__|, |__|</td><td>|__|__|__| (weeks)</td></tr>
<tr><td>2</td><td>|__|__|__|</td><td>________________</td><td>☐ Transit
☐ Stay → duration |__|__|__| (weeks)
→ accommodation** |__|, |__|, |__|, |__|</td><td>|__|__|__| (weeks)</td></tr>
<tr><td>3</td><td>|__|__|__|</td><td>________________</td><td>☐ Transit
☐ Stay → duration |__|__|__| (weeks)
→ accommodation** |__|, |__|, |__|, |__|</td><td>|__|__|__| (weeks)</td></tr>
<tr><td>4</td><td>|__|__|__|</td><td>________________</td><td>☐ Transit
☐ Stay → duration |__|__|__| (weeks)
→ accommodation** |__|, |__|, |__|, |__|</td><td>|__|__|__| (weeks)</td></tr>
<tr><td>5</td><td>|__|__|__|</td><td>________________</td><td>☐ Transit
☐ Stay → duration |__|__|__| (weeks)
→ accommodation** |__|, |__|, |__|, |__|</td><td>|__|__|__| (weeks)</td></tr>
<tr><td>6</td><td>|__|__|__|</td><td>________________</td><td>☐ Transit
☐ Stay → duration |__|__|__| (weeks)
→ accommodation** |__|, |__|, |__|, |__|</td><td>|__|__|__| (weeks)</td></tr>
<tr><td>7</td><td>|__|__|__|</td><td>________________</td><td>☐ Transit
☐ Stay → duration |__|__|__| (weeks)
→ accommodation** |__|, |__|, |__|, |__|</td><td>|__|__|__| (weeks)</td></tr>
<tr><td>8</td><td>|__|__|__|</td><td>________________</td><td>☐ Transit
☐ Stay → duration |__|__|__| (weeks)
→ accommodation** |__|, |__|, |__|, |__|</td><td>|__|__|__| (weeks)</td></tr>
<tr><td>9</td><td>|__|__|__|</td><td>________________</td><td>☐ Transit
☐ Stay → duration |__|__|__| (weeks)
→ accommodation** |__|, |__|, |__|, |__|</td><td>|__|__|__| (weeks)</td></tr>
<tr><td>10</td><td>|__|__|__|</td><td>________________</td><td>☐ Transit
☐ Stay → duration |__|__|__| (weeks)
→ accommodation** |__|, |__|, |__|, |__|</td><td>|__|__|__| (weeks)</td></tr>
</table>

** 1 – Boat; 2 – Bus/Car/Truck; 3 – Airplane; 4 – Train; 5 – On foot; 6 – Other*

*** 1 – Hotel/Pension; 2 – Family/friends; 3 – Hospitals/clinics; 4 – Detention centre/prison; 5 - Refugee camp; 6 – Homeless/living on the street; 7 – Homeless shelter; 8 – Other*

aida_TB	LMU-IMPH- AIDA-TB	Questionnaire	QUES	LMU KLINIKUM
	Version 3.8 (05-Jul-2017)	Participant-ID: |__|__| - |__|__|__|__| - |__|	Page 5 of 9	

2.5	Has the participant stayed in one of the following shelters >1 week at a time? *More than one entry allowed.*	☐<br>☐<br><br>☐<br>☐<br><br>☐<br><br>☐<br>☐<br>☐<br><br>☐<br>☐<br>☐	No<br>Detention centre/ prison |__|__|__| *(weeks)*<br>→If yes, specify ____________<br>Hospital/ clinics |__|__|__| *(weeks)*<br>Formal refugee camp |__|__|__| *(weeks)*<br>→If yes, specify ____________<br>Informal refugee camp |__|__|__| *(weeks)*<br>→If yes, specify ____________<br>Homeless shelter |__|__|__| *(weeks)*<br>Homeless |__|__|__| *(weeks)*<br>Other type of mass accommodation |__|__|__| *(weeks)*<br>→If yes, specify ____________<br>Don't know<br>Unwilling to disclose<br>N/A	
2.6	Has the participant used one of the following modes of travel for >24h at a time? *More than one entry allowed.*	☐<br>☐<br>☐<br>☐<br>☐<br>☐<br>☐	No<br>Bus/ Car/ Truck |__|__|__| *(days)*<br>Boat |__|__|__| *(days)*<br>Train |__|__|__| *(days)*<br>Don't know<br>Unwilling to disclose<br>N/A	
2.7	Has any chest X-ray been performed en route?	☐<br>☐<br>☐<br>☐<br>☐	No<br>Yes<br>Don't know<br>Unwilling to disclose<br>N/A	
**3.**	**Medical History**			
3.1	Does the participant have a history of respiratory complaints? *More than one entry allowed.*	☐<br>☐<br>☐<br>☐	No<br>Unwilling to disclose<br>Don`t know<br>N/A	
		☐	Yes ☐ Asthma<br>☐ Silicosis<br>☐ Interstitial lung disease<br>☐ COPD<br>☐ Lung cancer<br>☐ Other, specify ____________	
3.2	Does the participant have any chronic diseases? *More than one entry allowed.*	☐<br>☐<br>☐<br>☐	No<br>Unwilling to disclose<br>Don't know<br>N/A	
		☐	Yes ☐ Diabetes<br>☐ Kidney disease<br>☐ High blood pressure<br>☐ Other, specify ____________	

aida_TB	LMU-IMPH- AIDA-TB	Questionnaire	QUES	LMU KLINIKUM
	Version 3.8 (05-Jul-2017)	Participant-ID: |__|__| - |__|__|__|__| - |__|	Page 6 of 9	

3.3	Has the participant ever been diagnosed with HIV?	☐<br>☐<br>☐<br>☐<br>☐	No<br>Never been tested<br>Don`t know<br>Unwilling to disclose<br>N/A	
		☐	Yes Date of diagnosis |__|__|__| . |__|__|__|__|[1]<br>Month Year	
3.4	Does the participant take any chronic medication?	☐<br>☐<br>☐	No<br>Unwilling to disclose<br>N/A	Go to Q3.5
		☐	Yes	
	a) If yes, specify type of treatment: *More than one entry allowed.*	☐<br>☐<br>☐<br>☐	HIV treatment<br>Immunosuppressive treatment<br>Anti-diabetes<br>Other, specify: ____________	
	b) If yes, specify name and duration *More than one entry allowed.*		1. Name____________ |__|__|__| *(months)*<br>2. Name____________ |__|__|__| *(months)*<br>3. Name____________ |__|__|__| *(months)*<br>4. Name____________ |__|__|__| *(months)*<br>5. Name____________ |__|__|__| *(months)*<br>6. Name____________ |__|__|__| *(months)*	
3.5	Has the participant received any antibiotic treatment within the last 4 weeks?	☐<br>☐<br>☐<br>☐	No<br>Don't know<br>Unwilling to disclose<br>N/A	Go to Q4.1
		☐	Yes	
	a) If yes, specify name and duration: *More than one entry allowed.*		1. Name____________ |__|__|__| (days)<br>2. Name____________ |__|__|__| (days)<br>3. Name____________ |__|__|__| (days)<br>4. Name____________ |__|__|__| (days)	
**4.**	**TB and TB contacts**			
4.1	Has the participant ever been diagnosed or treated for TB?	☐<br>☐<br>☐<br>☐	No<br>Don`t know<br>Unwilling to disclose<br>N/A	Go to Q4.2
		☐	Yes |__|__|__|__| *(year)*	
	a) Has the participant ever been diagnosed with MDR TB?	☐<br>☐<br>☐<br>☐<br>☐	No<br>Don't know<br>Unwilling to disclose<br>N/A<br>Yes |__|__|__|__| *(year)* [1]	
	b) Has the participant ever been treated for TB?	☐<br>☐<br>☐	No<br>Don't know<br>Unwilling to disclose	Go to Q4.2

aida_TB	LMU-IMPH- AIDA-TB	Questionnaire	QUES	LMU KLINIKUM
	Version 3.8 (05-Jul-2017)	Participant-ID: |__|__| - |__|__|__|__| - |__|	Page 7 of 9	

		☐	NA	
		☐	Yes	
	c) Did he/she complete the treatment?	☐<br>☐<br>☐<br>☐	No<br>Don't know<br>Unwilling to disclose<br>N/A	
		☐	Yes	
	d) Specify drug and duration (weeks):<br>*More than one entry allowed.*	☐<br>☐<br>☐<br>☐<br>☐<br>☐	Rifampicin |__|__| *(weeks)*<br>Isoniazid |__|__| *(weeks)*<br>Pyrazinamide |__|__| *(weeks)*<br>Ethambutol |__|__| *(weeks)*<br>Other, specify ______________ |__|__| *(weeks)*<br>Drug name unknown |__|__| *(weeks)*	
	e) Specify how many months ago last dose was taken		|__|__|__| *(months)*	
4.2	What was the reason for the current admission or testing for TB?<br>*More than one entry allowed.*	☐<br><br><br><br>☐<br>☐<br><br><br><br><br><br>☐<br>☐<br>☐	Symptoms: ☐ Cough >2 weeks/dyspnoea<br>☐ Haemoptysis<br>☐ Fatigue/night sweat<br>☐ Other: ________________<br>Contact tracing<br>Admission examination ☐ Entry screening (asylum procedures)<br>☐ Shared accommodation<br>☐ Detention centre/prison<br>☐ Homeless shelter<br>☐ Homeless/living on the street<br>☐ Other: ________________<br>Don't know<br>Unwilling to disclose<br>N/A	
4.3	Has the participant been vaccinated with BCG?	☐<br>☐<br>☐<br>☐<br>☐	No<br>Don't know<br>Unwilling to disclose<br>N/A<br>Yes	
**5.**	**Exposure to potential TB cases**			
5.1	Are family members/ contacts experiencing respiratory complaints (chronic cough)?	☐<br>☐<br>☐<br>☐<br>☐	No<br>Don't know<br>Unwilling to disclose<br>N/A<br>Yes, duration |__|__|__| *(weeks)*	

aida_TB	LMU-IMPH- AIDA-TB	Questionnaire	QUES	LMU KLINIKUM
	Version 3.8 (05-Jul-2017)	Participant-ID: |__|__| - |__|__|__|__| - |__|	Page 8 of 9	

5.2	Has the participant ever had contact to a person with known TB?	☐<br>☐<br>☐<br>☐	No<br>Don't know<br>Unwilling to disclose<br>N/A	Go to Q6.1
		☐	Yes, specify year |__|__|__|__|	
	a) What is the relation to this person?<br>*More than one entry allowed.*	☐<br>☐<br>☐<br>☐<br>☐<br>☐	Mother<br>Father<br>Child<br>Sibling<br>Husband/Wife/Partner<br>Other, specify ________________	
	b) Does or did the participant live in the same household?	☐<br>☐<br>☐<br>☐	No<br>Unwilling to disclose<br>N/A<br>Yes	
	c) Does or did the participant sleep in the same room?	☐<br>☐<br>☐<br>☐	No<br>Unwilling to disclose<br>N/A<br>Yes → If Yes, does the participant sleep in the same bed?<br>☐ No<br>☐ Yes<br>☐ Unwilling to disclose<br>☐ N/A	
**6.**	**Exposure to other TB risk factors**			
6.1	Has the participant ever worked in a mine?	☐<br>☐<br>☐<br>☐	No<br>Unwilling to disclose<br>N/A<br>Yes	
6.2	Has the participant ever been admitted to a detention centre/prison?	☐<br>☐<br>☐<br>☐	No<br>Unwilling to disclose<br>N/A<br>Yes	
6.3	Did the participant make use of indoor cooking with solid fuels at home at last residence, recently (in the last 5 years)?<br>*(e.g. wood, animal dung, coal. Heating with solid fuel is not meant by this)*			

aida_TB	LMU-IMPH- AIDA-TB	Questionnaire	QUES	LMU KLINIKUM
	Version 3.8 (05-Jul-2017)	Participant-ID: |__|__| - |__|__|__|__| - |__|	Page 9 of 9	

6.4	Does the participant smoke tobacco?<br>*More than one entry allowed.*<br>*If quantity is unknown, insert 99; if NA, insert 0.*	☐<br>☐<br>☐	No<br>Unwilling to disclose<br>N/A	
		☐	Yes |__|__| *(years)*<br>Number of cigarettes : |__|__|/day<br>Number of cigars : |__|__|/day<br>Number of pipes : |__|__|/day	
6.5	Does the participant drink alcohol?<br>*More than one entry allowed.*<br>*If quantity is unknown, insert 99; if NA, insert 0.*	☐<br>☐<br>☐	No<br>Unwilling to disclose<br>N/A	
		☐	Yes |__|__| years<br>Shots of Liquor: |__|__|/week<br>Number of beer: |__|__|/week<br>Glasses of wine |__|__|/week<br>Other: ____________ |__|__|/week	
6.6	Does the participant use illegal drugs?<br>*If quantity is unknown, insert 99; if NA, insert 0.*	☐<br>☐<br>☐	No<br>Unwilling to disclose<br>N/A	
		☐	Yes |__|__| years, times per week |__|__|	

Study clinician's/Designee's Initials:

(1) Day (DD) - Month (MMM) – Year (YYYY); Enter 99 if day is unknown; 999 if month is unknown; 9999 if year is unknown

Supplemental figure 1:

Patients were classified as having:

- **Confirmed TB**
  - MTB culture and/or MTB complex positive
  - PCR and/or GeneXpert Ultra, if more than trace and if no previous TB, positive
- **Unconfirmed TB**
  - MTB culture and/or MTB complex negative
  - PCR negative
  - TB treatment was initiated based on clinical findings
- **No TB**
  - Healthy
    - With or without latent infection (=IGRA positive)
  - History of TB disease
  - Definite other disease
- **Unclassifiable**
  - Unclear, no sufficient data

Supplemental table 1: Demographic characteristics of children by citizenship

		German citizens (n=18)	EU citizen (n=4)	Non-EU citizen (n=21)	All (n=43)
Gender					
Female	n (%)	10 (55.6)	2 (50.0)	7 (33.3)	19 (44.2)
Male	n (%)	8 (44.4)	2 (50.0)	14 (66.7)	24 (55.8)
Age category					
Age in years	median (IQR)	3.0 (2.0 - 6.0)	7.5 (3.5 - 13.5)	9.0 (2.0 - 15.0)	5.0 (2.0 - 14.0)
<2 years	n (%)	7 (38.8)	1 (25.0)	7 (33.3)	15 (34.9)
2-<5 years	n (%)	6 (33.3)	1 (25.0)	3 (14.3)	10 (23.3)
5-<15 years	n (%)	4 (22.2)	1 (25.0)	5 (23.8)	10 (23.3)
15-<18 years	n (%)	1 (5.5)	1 (25.0)	6 (28.6)	8 (18.6)
Migrant status					
Migrant	n (%)	10 (55.6)	4 (100.0)	11 (52.4)	25 (58.1)
Refugee	n (%)	0 (0.0)	0 (0.0)	10 (47.6)	10 (23.3)
Time in Germany (years)	median (IQR)	3.0 (2.0 - 7.0)	5.0 (3.5 - 8.5)	2.0 (1.0 - 2.0)	2.0 (1.0 - 5.0)
Reason for testing					
Symptoms	n (%)	1 (5.9)	1 (25.0)	5 (23.8)	7 (16.7)
Contact	n (%)	13 (76.5)	3 (75.0)	10 (47.6)	26 (61.9)
Admission examination	n (%)	1 (1.5)	1 (5.9)	0 (0.0)	6 (28.6)
Symptoms & Contact	n (%)	1 (1.5)	1 (5.9)	0 (0.0)	0 (0.0)
TB status					
Confirmed TB	n (%)	0 (0.0)	1 (25.0)	5 (23.8)	6 (14.0)
Unconfirmed TB	n (%)	3 (16.7)	1 (25.0)	3 (14.3)	7 (16.3)
No TB	n (%)	15 (83.3)	2 (50.0)	13 (61.9)	30 (69.8)

Migrant= participants whose birth country was not Germany (1stgeneration) or whose parents were born outside of Germany (2nd generation)
Refugee= participants who completed our migration questionnaire

Supplemental table 2: TB status: TB findings in children with TB disease (confirmed + unconfirmed TB) by citizenship					
		German citizen (n=3)	EU citizen (n=2)	Non-EU citizen (n=8)	All (n=13)
TB confirmation status					
Confirmed TB	n (%)	0 (0.0)	1 (50.0)	5 (62.5.0)	6 (46.2)
TB microbiological findings					
LJ positive	n (%)	0 (0.0)	0 (0.0)	2 (25.0)	2 (15.4)
MGIT positive	n (%)	0 (0.0)	1 (50.0)	5 (62.5)	6 (46.2)
PCR positive	n (%)	0 (0.0)	0 (0.0)	4 (50.0)	4 (30.8)
Ultra positive	n (%)	0 (0.0)	1 (50.0)	1 (12.5)	2 (15.4)
TB immunological testing					
TST positive	n (%)	3 (100.0)	1 (50.0)	0 (0.0)	4 (30.8)
IGRA positive	n (%)	2 (66.7)	2 (100.0)	5 (62.5)	9 (69.2)

Supplemental table 3: Extent of TB disease & Risk factors by Reason for testing							
		Symptoms (n=191)	Contact (n=37)	Admission examination (n=32)	Symptoms + Contact (n=7)	Symptoms + Admission (n=10)	All (n=277)
TB status							
Confirmed TB	n (%)	114 (59.7)	6 (16.2)	9 (28.1)	4 (57.1)	5 (50.0)	138 (49.8)
Unconfirmed TB	n (%)	20 (10.5)	5 (13.5)	7 (21.9)	1 (14.3)	3 (30.0)	36 (13.0)
No TB	n (%)	57 (29.8)	26 (70.3)	16 (50.0)	2 (28.6)	2 (20.0)	103 (37.2)
TB microbiological findings							
LJ positive	n (%)	90 (47.1)	4 (10.8)	3 (9.4)	4 (57.1)	1 (10.0)	102 (36.8)
MGIT positive	n (%)	114 (59.7)	6 (16.2)	9 (28.1)	4 (57.1)	4 (40.0)	137 (49.5)
PCR positive	n (%)	88 (46.1)	2 (5.4)	3 (9.4)	4 (57.1)	4 (40.0)	101 (36.5)
Ultra positive	n (%)	52 (27.2)	1 (2.7)	1 (3.1)	3 (42.9)	1 (10.0)	58 (20.9)
TB immunological testing							
TST positive	n (%)	2 (1.0)	10 (27.0)	0 (0.0)	0 (0.0)	0 (0.0)	12 (4.3)
IGRA positive	n (%)	109 (57.1)	14 (37.8)	18 (56.3)	5 (71.4)	9 (90.0)	155 (56.0)
TB disease site							
Pulmonary TB	n (%)	94 (49.2)	10 (27.0)	14 (43.8)	5 (71.4)	7 (70.0)	130 (46.9)
Extrapulmonary TB (EPT)	n (%)	14 (7.3)	0 (0.0)	1 (3.1)	0 (0.0)	1 (10.0)	16 (5.8)
Pulmonary & EPT	n (%)	26 (13.6)	1 (2.7)	1 (3.1)	0 (0.0)	0 (0.0)	28 (10.1)
CXR findings							
Ralph score	median (IQR)	12.5 (5.0 - 50.0)	0.0 (0.0 - 5.0)	5.0 (2.0 - 8.0)	5.0 (3.0 - 50.0)	4.0 (0.0 - 6.5)	8.0 (3.0 - 30.0)
CXR finding abnormal (not specified)	n (%)	146 (76.4)	15 (40.5)	28 (87.5)	6 (85.7)	9 (90.0)	204 (73.6)
Symptomology							
Cough	n (%)	139 (72.8)	5 (13.5)	2 (6.3)	6 (85.7)	3 (30.0)	155 (56.0)
Haemoptysis	n (%)	29 (15.2)	0 (0.0)	0 (0.0)	1 (14.3)	1 (10.0)	31 (11.2)
Fever	n (%)	43 (22.5)	1 (2.7)	2 (6.3)	0 (0.0)	1 (10.0)	47 (17.0)
Loss of weight	n (%)	86 (45.0)	1 (2.7)	1 (3.1)	0 (0.0)	3 (30.0)	91 (32.9)
Other lab findings							
CRP (mg/l)	median (IQR)	18.7 (2.8 - 54.7)	1.0 (0.1 - 2.9)	1.5 (1.0 - 3.7)	4.6 (2.2 - 13.4)	5.5 (1.9 - 7.8)	6.9 (1.5 - 34.7)
CRP >= 5mg/l	n (%)	132 (69.8)	4 (12.9)	3 (9.4)	3 (42.9)	5 (50.0)	147 (54.6)
Vitamin D levels	median (IQR)	10.0 (6.0 - 22.0)	11.0 (5.5 - 19.0)	11.5 (7.0 - 17.0)	10.5 (7.0 - 21.0)	10.5 (6.0 - 15.0)	10.0 (6.0 - 20.0)
Vitamin D deficiency	n (%)	89 (46.6)	9 (24.3)	21 (65.6)	4 (57.1)	7 (70.0)	130 (46.9)
TB risk factors							
Previous TB	n (%)	20 (10.5)	1 (2.7)	5 (15.6)	1 (14.3)	0 (0.0)	27 (9.7)
TB contact	n (%)	43 (22.5)	37 (100.0)	3 (9.4)	7 (100.0)	0 (0.0)	90 (32.5)

Supplemental table 4: Clinical characteristics and general risk factors by TB status in Germans

		TB disease (n=28)	No TB (n=39)	All (n=67)
CXR findings				
Ralph score	median (IQR)	8.0 (5.0 - 20.0)	12.5 (5.0 - 60.0)	10.0 (5.0 - 50.0)
CXR finding abnormal (not specified)	n (%)	25 (89.3)	20 (51.3)	45 (67.2)
Symptomology				
Cough	n (%)	19 (67.9)	22 (56.4)	41 (61.2)
Haemoptysis	n (%)	2 (7.1)	1 (2.6)	3 (4.5)
Fever	n (%)	6 (21.4)	4 (10.3)	10 (14.9)
Loss of weight	n (%)	9 (32.1)	14 (35.9)	23 (34.3)
Other lab findings				
CRP (mg/l)	median (IQR)	16.2 (5.0 - 67.6)	2.4 (1.0 - 17.9)	7.5 (1.1 - 50.6)
CRP >= 5mg/l	n (%)	21 (77.8)	15 (40.5)	36 (56.3)
Vitamin D levels	median (IQR)	15.0 (8.0 - 28.0)	21.0 (14.0 - 39.0)	20.0 (10.0 - 34.0)
Vitamin D deficiency (<10ng/l)	n (%)	7 (25.0)	4 (10.3)	11 (16.4)
TB risk factors				
Previous TB	n (%)	2 (7.1)	5 (12.8)	7 (10.4)
TB contact	n (%)	8 (28.6)	15 (38.5)	23 (34.3)
Comorbidities				
HIV	n (%)	1 (3.6)	0 (0.0)	1 (1.5)
BMI	mean (SD)	22.7 (4.0)	19.2 (5.5)	20.5 (5.3)
Underweight	n (%)	1 (3.6)	16 (41.0)	17 (25.4)
Chronic respiratory diseases	n (%)	1 (3.6)	10 (25.6)	11 (16.4)
Cardiovascular disease	n (%)	10 (35.7)	7 (17.9)	17 (25.4)
Diabetes	n (%)	0 (0.0)	2 (5.1)	2 (3.0)
Tumor/oncologic disease	n (%)	1 (3.6)	1 (2.6)	2 (3.0)
Hepatitis B	n (%)	0 (0.0)	1 (2.6)	1 (1.5)
Other risk factors				
Mine work	n (%)	0 (0.0)	0 (0.0)	0 (0.0)
Indoor cooking (open fire)	n (%)	0 (0.0)	0 (0.0)	0 (0.0)
Smoking	n (%)	7 (25.0)	13 (33.3)	20 (29.9)
Illegal drugs	n (%)	1 (3.6)	0 (0.0)	1 (1.5)
Alcohol abuse	n (%)	7 (25.0)	9 (23.1)	16 (23.9)

Supplemental table 5: Clinical characteristics and general risk factors by TB status in EU citizens

		TB disease (n=28)	No TB (n=6)	All (n=34)
CXR findings				
Ralph score	median (IQR)	15.0 (5.0 - 55.0)	0.0 (0.0 - 55.0)	12.5 (5.0 - 55.0)
CXR finding abnormal (not specified)	n (%)	20 (71.4)	3 (50.0)	23 (67.6)
Symptomology				
Cough	n (%)	19 (67.9)	2 (33.3)	21 (61.8)
Haemoptysis	n (%)	1 (3.6)	0 (0.0)	1 (2.9)
Fever	n (%)	6 (21.4)	1 (16.7)	7 (20.6)
Loss of weight	n (%)	12 (42.9)	2 (33.3)	14 (41.2)
Other lab findings				
CRP (mg/l)	median (IQR)	11.5 (3.8 - 48.1)	8.5 (0.0 - 65.7)	11.0 (3.0 - 50.3)
CRP >= 5mg/l	n (%)	19 (67.9)	3 (50.0)	22 (64.7)
Vitamin D levels	median (IQR)	11.0 (8.0 - 23.0)	11.0 (11.0 - 15.0)	11.0 (8.0 - 21.0)
Vitamin D deficiency (<10ng/l)	n (%)	8 (28.6)	0 (0.0)	8 (23.5)
TB risk factors				
Previous TB	n (%)	0 (0.0)	0 (0.0)	0 (0.0)
TB contact	n (%)	13 (46.4)	2 (33.3)	15 (44.1)
Comorbidities				
HIV	n (%)	0 (0.0)	0 (0.0)	0 (0.0)
BMI	mean (SD)	21.6 (3.5)	22.5 (5.0)	21.8 (3.7)
Underweight	n (%)	2 (7.1)	1 (16.7)	3 (8.8)
Chronic respiratory diseases	n (%)	2 (7.1)	1 (16.7)	3 (8.8)
Cardiovascular disease	n (%)	2 (7.1)	0 (0.0)	2 (5.9)
Diabetes	n (%)	4 (14.3)	0 (0.0)	4 (11.8)
Tumor/oncologic disease	n (%)	1 (3.6)	0 (0.0)	1 (2.9)
Hepatitis B	n (%)	1 (3.6)	0 (0.0)	1 (2.9)
Other risk factors				
Mine work	n (%)	0 (0.0)	0 (0.0)	0 (0.0)
Indoor cooking (open fire)	n (%)	0 (0.0)	0 (0.0)	0 (0.0)
Smoking	n (%)	17 (60.7)	4 (66.7)	21 (61.8)
Illegal drugs	n (%)	0 (0.0)	0 (0.0)	0 (0.0)
Alcohol abuse	n (%)	9 (32.1)	2 (33.3)	11 (32.4)

Supplementary table 6: Clinical characteristics and general risk factors by TB status in non-EU citizens

		TB disease (n=121)	No TB (n=62)	All (n=183)
CXR findings				
Ralph score	median (IQR)	8.0 (3.0 - 30.0)	3.0 (0.0 - 10.0)	5.0 (0.0 - 20.0)
CXR finding abnormal (not specified)	n (%)	102 (84.3)	38 (61.3)	140 (76.5)
Symptomology				
Cough	n (%)	70 (57.9)	25 (40.3)	95 (51.9)
Haemoptysis	n (%)	19 (15.7)	9 (14.5)	28 (15.3)
Fever	n (%)	20 (16.5)	11 (17.7)	31 (16.9)
Loss of weight	n (%)	44 (36.4)	12 (19.4)	56 (30.6)
Other lab findings				
CRP (mg/l)	median (IQR)	15.7 (3.4 - 42.2)	1.3 (0.9 - 3.3)	6.0 (1.5 - 26.6)
CRP >= 5mg/l	n (%)	81 (66.9)	11 (19.3)	92 (51.7)
Vitamin D levels	median (IQR)	8.0 (5.0 - 12.0)	12.0 (7.0 - 20.0)	9.0 (6.0 - 14.0)
Vitamin D deficiency (<10ng/l)	n (%)	69 (38.8)	18 (51.6)	87 (43.2)
TB risk factors				
Previous TB	n (%)	9 (7.4)	12 (19.4)	21 (11.5)
TB contact	n (%)	36 (29.8)	19 (30.6)	55 (30.1)
Comorbidities				
HIV	n (%)	2 (1.7)	3 (4.8)	5 (2.7)
BMI	mean (SD)	22.2 (4.2)	22.3 (6.1)	22.2 (4.9)
Underweight	n (%)	18 (14.9)	13 (21.0)	31 (16.9)
Chronic respiratory diseases	n (%)	5 (4.1)	4 (6.5)	9 (4.9)
Cardiovascular disease	n (%)	10 (8.3)	3 (4.8)	13 (7.1)
Diabetes	n (%)	4 (3.3)	1 (1.6)	5 (2.7)
Tumor/oncologic disease	n (%)	1 (0.8)	1 (1.6)	2 (1.1)
Hepatitis B	n (%)	3 (2.5)	1 (1.6)	4 (2.2)
Other risk factors				
Mine work	n (%)	2 (1.7)	1 (1.6)	3 (1.6)
Indoor cooking (open fire)	n (%)	19 (15.7)	6 (9.7)	25 (13.7)
Smoking	n (%)	37 (30.6)	18 (29.0)	55 (30.1)
Illegal drugs	n (%)	2 (1.7)	1 (1.6)	3 (1.6)
Alcohol abuse	n (%)	22 (18.2)	11 (17.7)	33 (18.0)

Supplementary table 7: Clinical characteristics and risk factors by TB status in children		TB disease (n=13)	No TB (n=30)	All (n=43)
CXR findings				
Ralph score	median (IQR)	5.0 (3.0 - 20.0)	0.0 (0.0 - 0.0)	0.0 (0.0 - 5.0)
CXR finding abnormal (not specified)	n (%)	12 (92.3)	4 (13.3)	16 (37.2)
Symptomology				
Cough	n (%)	2 (15.4)	5 (16.7)	7 (16.3)
Haemoptysis	n (%)	1 (7.7)	2 (6.7)	3 (7.0)
Fever	n (%)	2 (15.4)	1 (3.3)	3 (7.0)
Loss of weight	n (%)	4 (30.8)	1 (3.3)	5 (11.6)
Other lab findings				
CRP (mg/l)	median (IQR)	6.5 (3.5 - 20.2)	1.0 (0.1 - 1.0)	1.0 (0.2 - 4.6)
CRP >= 5mg/l	n (%)	7 (58.3)	2 (8.0)	9 (24.3)
Vitamin D levels	median (IQR)	13.5 (8.5 - 21.0)	13.5 (11.0 - 20.0)	13.5 (10.0 - 21.0)
Vitamin D deficiency (<10ng/l)	n (%)	6 (46.2)	6 (20.0)	12 (27.9)
TB risk factors				
Previous TB	n (%)	1 (7.7)	1 (3.3)	2 (4.7)
TB contact	n (%)	5 (38.5)	25 (83.3)	30 (69.8)
Comorbidities				
HIV	n (%)	0 (0.0)	0 (0.0)	0 (0.0)
BMI	mean (SD)	19.1 (2.2)	16.2 (2.5)	17.0 (2.7)
Other risk factors				
Indoor cooking (open fire)	n (%)	1 (7.7)	0 (0.0)	1 (2.3)
Smoking	n (%)	2 (15.4)	1 (3.3)	3 (7.0)
Alcohol abuse	n (%)	1 (7.7)	0 (0.0)	1 (2.3)

Supplemental table 8: Association of various factors with TB disease (N = 117; TB disease = 79; % TB disease = 67.5) in the refugee cohort

Covariate	N	TB disease	% TB disease	RR	Univariable 95% CI	p-value	RR	Multivariable 95% CI	p-value
Sex									
f*	15	11	73,3	1,00	-	-	1,00	-	-
m	61	39	63,9	0,87	0.61 - 1.25	0.4621	0,93	0.67 - 1.29	0.6658
Age									
per year	-	-	-	1,00	0.99 - 1.02	0.6769	1,01	0.99 - 1.02	0.5603
Flight routes									
West Africa*	19	9	47,4	1,00	-	-	1,00	-	-
East Africa	36	26	72,2	1,54	0.91 - 2.62	0.1077	1,53	0.90 - 2.60	0.1163
Asia	21	15	71,4	1,50	0.87 - 2.61	0.1462	1,38	0.80 - 2.40	0.2519
Flight duration									
Per month	-	-	-	0,99	0.97 - 1.01	0.2011	0,99	0.97 - 1.01	0.2468

N = number of observations; TB disease = number of TB cases; % TB disease = (N-events/N) *100; RR = risk ratio; 95% CI = 95% confidence interval
* reference stratum
Uni- and multi-variable Poisson regression results; using robust variance estimates
"Uni-variable" model(s) adjusted for: Sex; Age
